# Clinical effects of constant rate infusions of medetomidine–propofol combined with sevoflurane anesthesia in Thoroughbred racehorses undergoing arthroscopic surgery

**DOI:** 10.1186/s13028-018-0426-0

**Published:** 2018-11-05

**Authors:** Hirotaka Tokushige, Atsushi Okano, Daisuke Arima, Hideki Ito, Yoshinori Kambayashi, Yohei Minamijima, Minoru Ohta

**Affiliations:** 1 Racehorse Clinic, Miho Training Center, Japan Racing Association, 2500-2 Mikoma, Miho-mura, Inashiki-gun, Ibaraki 300-0493 Japan; 2 Analytical Chemistry Section, Laboratory of Racing Chemistry, 1731-2 Tokami-cyo, Utsunomiya-shi, Tochigi 320-0851 Japan; 3 Equine Research Institute, Japan Racing Association, 1400-4 Shiba, Shimotsuke, Tochigi 329-0412 Japan

**Keywords:** Constant rate infusion, Medetomidine, Propofol, Racehorse, Sevoflurane

## Abstract

**Background:**

The aim of the present study was to evaluate clinical efficacy of constant rate infusions (CRIs) of medetomidine–propofol combined with sevoflurane anesthesia in Thoroughbred racehorses undergoing arthroscopic surgery. Thirty horses were sedated intravenously (IV) with medetomidine (6.0 μg/kg) and midazolam (0.02 mg/kg) and induced IV with ketamine (1.0 mg/kg) and propofol (1.0 mg/kg). These horses were randomly allocated to three groups and maintained with sevoflurane and CRI of either medetomidine (3.0 μg/kg/h) (Group M; n = 10); or medetomidine (3.0 μg/kg/h) and propofol (3.0 mg/kg/h) (Group MP3; n = 10); or medetomidine (3.0 μg/kg/h) and propofol (6.0 mg/kg/h) (Group MP6; n = 10). End-tidal sevoflurane concentration (ET_SEVO_), cardiovascular parameters, plasma propofol concentration, and recovery time and quality were compared among groups. Data were analyzed by using ANOVA with Tukey’s multiple comparison test, considering *P *< 0.05 significant.

**Results:**

ET_SEVO_ (%) was 2.4 ± 0.1 in Group M, 1.7 ± 0.2 in Group MP3, and 1.4 ± 0.2 in Group MP6; ET_SEVO_ declined significantly in a propofol-dose-dependent manner. The rates of dobutamine infusion (μg/kg/min) required to keep the mean arterial blood pressure over 70 mmHg were significantly lower in Group MP3 (0.20 ± 0.10) and Group MP6 (0.15 ± 0.06) than in Group M (0.37 ± 0.18). Recovery time and quality did not differ among groups. All horses in Group MP3 required only one attempt to stand, and recovery quality was excellent. Plasma propofol concentrations were stable throughout maintenance of anesthesia in Group MP3, whereas those in Group MP6 increased significantly with increasing duration of maintenance.

**Conclusions:**

CRIs of medetomidine–propofol reduced the sevoflurane requirement for surgical anesthesia as the propofol dose increased, compared with a CRI of medetomidine alone. Additionally, the two propofol protocols provided good maintenance of cardiovascular function. CRIs of medetomidine (3.0 μg/kg/h) and propofol (3.0 mg/kg/h) resulted in excellent-quality recovery. This protocol could therefore be an especially useful additive to sevoflurane anesthesia in Thoroughbred racehorses undergoing arthroscopic surgery.

## Background

Sevoflurane has the advantages of rapid induction, easy control of anesthetic depth and rapid recovery because of its low blood solubility [1–3]. However, sevoflurane is known to induce dose-dependent cardiopulmonary depression, which increases the risk of peri-anesthetic mortality and death [4–6]. Therefore, in equine practice, balanced anesthesia is usually applied to decrease the requirement of inhalant anesthetics and thus limit their cardiovascular depressant effects [7].

The short half-life of medetomidine and its selectivity and potency make it suitable for use as a constant rate infusion (CRI) for balanced anesthesia in horses [8–11]. In our previous study, we found that medetomidine CRI (3.0 µg/kg/h) reduced the sevoflurane requirement during arthroscopic surgery by approximately 10% in Thoroughbred racehorses, resulting in good maintenance of cardiopulmonary function and an improvement in the quality of recovery from anesthesia [12]. However, the anesthetic sparing effect of medetomidine CRI on sevoflurane was insufficient to fully minimize cardiac depression, and cardiovascular depression during the maintenance period still remained a concern.

Propofol has been described as an intravenous (IV) anesthetic suitable for total intravenous anesthesia (TIVA) lasting > 2 h because of its short half-life and the resultant rapid and good recovery [13, 14]. In a previous report, TIVA with propofol was suggested to be suitable for long-term anesthesia in horses because the degree of cardiovascular depression was less than inhalation anesthesia [15]. Medetomidine–propofol TIVA has been successfully employed with good cardiovascular function and anesthetic recovery in a wide range of surgical procedures in horses [16]. Additionally, the use of propofol with inhalational anesthesia has been reported in horses [17, 18]. Villalba et al. [18] demonstrated that medetomidine–propofol CRI reduced the minimum alveolar concentration (MAC) of isoflurane in experimental horses, improved the stability of arterial blood pressure, and resulted in a good-quality recovery. However, to the best of our knowledge, the sevoflurane requirement during CRIs of medetomidine–propofol and the adequate propofol infusion rate when propofol is combined with inhalational anesthetic agents under surgical procedures have not been investigated in clinical cases. We hypothesized that CRIs of medetomidine–propofol would result in a useful decrease in sevoflurane requirement in relation to the rate of propofol CRI, and that this decrease would be associated with good cardiovascular function and a good-quality recovery.

The purpose of this study was to evaluate the clinical effects of CRIs of medetomidine–propofol combined with sevoflurane anesthesia in Thoroughbred racehorses undergoing arthroscopic surgery.

## Methods

### Horses

Thirty Thoroughbred racehorses with chip fractures of the carpal bones were included in this study. Informed consent was obtained from the owner and the trainer before arthroscopic surgery. The surgery was performed on a single leg or both legs by experienced surgeons. All horses were subjected to preanesthetic electrocardiography, blood biochemistry and hematology on the day before surgery. Food, but not water, was withheld for 12 h prior to anesthesia.

### Experimental protocol

All horses were premedicated with medetomidine (Dorbene; Vetcare Oy, Salo, Finland) (6.0 μg/kg, IV) and midazolam (Dormicum; Astellas Pharma Inc., Tokyo, Japan) (20 μg/kg, IV) together. Anesthesia was induced by injection of 1% propofol (Propofol 1%; Nichi-Iko Co., Ltd., Toyama, Japan) (1.0 mg/kg, IV) and ketamine (Ketalar; Daiichi-Sankyo Co., Ltd., Tokyo, Japan) (1.0 mg/kg, IV) in three groups. After induction of anesthesia, the horses were intubated endotracheally and positioned in dorsal recumbency. Anesthesia was maintained with sevoflurane (Sevofrane; Maruishi Pharmaceutical Co., Ltd., Osaka, Japan) and oxygen (approximately 5 L/min) to produce a surgical plane of anesthesia. The horses were connected to a circle system and intermittent positive pressure ventilation initiated (MOK 94; Silver Medical Co., Tokyo, Japan) with a peak airway pressure of 25 cmH_2_O. The ventilator settings were chosen to maintain the arterial carbon dioxide tension (PaCO_2_) between 45 and 55 mmHg. Lactated Ringer’s solution was administered at a rate of approximately 10 mL/kg/h throughout anesthesia.

Horses were randomly assigned to one of three groups and received either medetomidine infused IV at 3.0 μg/kg/h throughout maintenance (Group M) (10 horses); medetomidine IV at 3.0 μg/kg/h plus propofol IV at 3.0 mg/kg/h (Group MP3) (10 horses); or medetomidine IV at 3.0 μg/kg/h plus propofol IV at 6.0 mg/kg/h (Group MP6) (10 horses).

A base-apex lead electrocardiogram was used to monitor heart rate (HR) and rhythm. A 20-G catheter was placed in the facial artery for measurement of arterial blood pressure and for arterial blood sample collection after connection to the breathing circuit. Respiratory gas was collected continuously, and the end-tidal sevoflurane concentration (ET_SEVO_) was determined by infrared absorption. The ET_SEVO_ was recorded throughout anesthesia, and HR, systolic arterial blood pressure (SAP), diastolic arterial blood pressure (DAP), and mean arterial blood pressure (MAP) were recorded every 5 min by an anesthesia monitoring system (BP608; Omron Colin Co., Ltd., Tokyo, Japan). Arterial blood samples were collected every 15 min and arterial carbon dioxide partial pressure (PaCO_2_), arterial oxygen partial pressure (PaO_2_) and pH were immediately analyzed by a blood-gas analyzer (ABL800 FLEX; Radiometer Co., Ltd., Tokyo, Japan). Hypotension was defined as MAP < 70 mmHg and corrected with dobutamine (Dobutrex; Shionogi & Co., Ltd., Osaka, Japan) infusion. The vaporizer setting of sevoflurane was based on observation of standard clinical signs to achieve a surgical plane of anesthesia. Anesthetic depth was judged to be light, if movement, brisk palpebral response, spontaneous nystagmus or sudden changes in arterial blood pressure and HR were observed.

Immediately after end of sevoflurane anesthesia, horses in all three groups were transported to the recovery room. They were allowed to recover without assistance and were given no additional sedatives. Oxygen was supplied via the endotracheal tube using a demand valve at one to two breaths per minute until spontaneous ventilation resumed. Extubation was performed once spontaneous ventilation resumed but prior to swallowing. The recovery phases were continuously monitored by use of a wide-angle high-resolution camera located at 3 m high on the wall of the recovery room. On the basis of these images, the quality of recovery was subjectively assessed by experienced anesthetists who were unaware of drug given using a scoring of 1–5 (1: poor; 2: marginal; 3: fair; 4: good; 5: excellent) [19]. The number of attempts to stand and the times taken from the end of anesthesia to the appearance of spontaneous respiration, extubation, first movement, sternal recumbency, first attempt to stand, and standing were recorded.

### Plasma propofol analysis

Blood samples were collected from all horses in Groups MP3 and MP6 after induction of anesthesia; 15, 30, and 45 min after connection to the breathing circuit; and immediately after standing. All blood samples were immediately placed on ice, and then the plasma was separated from the blood and frozen at − 20 °C. Propofol in plasma was extracted by liquid–liquid extraction using methyl tert-butyl ether. The extracted substance was analyzed by using liquid chromatography–tandem mass spectrometry with a Shimadzu Prominence HPLC system (Shimadzu Co., Tokyo, Japan) and AB Sciex QTRAP 4000 mass spectrometer (AB Sciex, Framingham, MA, USA).

### Statistical analysis

Data on age, body weight, mean ET_SEVO_, mean dobutamine infusion rate, blood gases, duration of anesthesia, and recovery times were tested for statistically significant differences between groups by using one-way analysis of variance (ANOVA). If significant differences were found, a Tukey multiple comparison test was conducted. Data on repeatedly measured variables (cardiovascular parameters in the three groups and plasma propofol concentrations in Groups MP3 and MP6) were tested for significant differences among and within groups by using repeated measures ANOVA. If significant differences were found, a post hoc analysis (pair-wise Tukey multiple comparison test) was conducted. Values are given as means ± deviation (SD). Data on the number of attempts to stand and recovery scores were tested for significant differences among groups by using Kruskal–Wallis one-way ANOVA with a Dunn’s post hoc test. Statistical significance was set at P < 0.05.

## Results

**Fig. 1:**
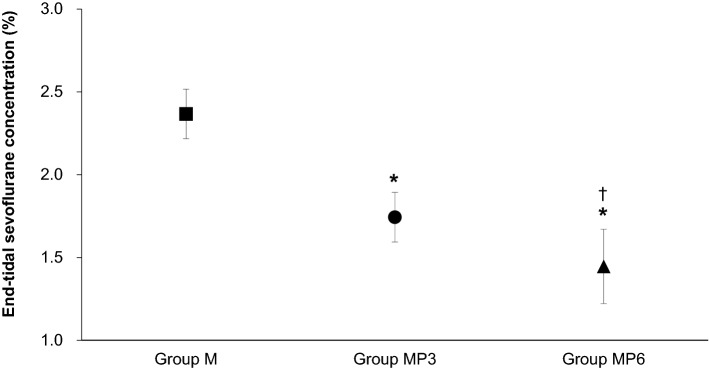
Mean end-tidal sevoflurane concentration during maintenance of anesthesia. Horses were maintained with sevoflurane and CRI of either medetomidine (3.0 μg/kg/h) (Group M; n = 10); or medetomidine (3.0 μg/kg/h) and propofol (3.0 mg/kg/h) (Group MP3; n = 10); or medetomidine (3.0 μg/kg/h) and propofol (6.0 mg/kg/h) (Group MP6; n = 10). *Significant difference compared with Group M (P < 0.001). ^†^Significant difference compared with Group MP3 (P = 0.003)

No abnormalities were found in any horses in the results of preanesthetic blood examination and electrocardiography. The horses in all three groups recovered uneventfully, without post-operative complications. The mean ± SD age and body weight were 2.7 ± 0.5 years and 445 ± 32 kg in Group M, 3.1 ± 0.9 years and 450 ± 17 kg in Group MP3, and 3.2 ± 0.6 years and 463 ± 26 kg in Group MP6. There were no significant differences in age and body weight among the three groups. Durations of anesthesia in Group M, Group MP3, and Group MP6 were 59 ± 9 min, 65 ± 17 min, and 70 ± 20 min; these values did not differ significantly. The ET_SEVO_ values during the maintenance period are shown in Fig. 1. The ET_SEVO_ values (%) were 2.4 ± 0.1 in Group M, 1.7 ± 0.2 in Group MP3, and 1.4 ± 0.2 in Group MP6. The ET_SEVO_ values in Group MP3 and Group MP6 were significantly lower than those in Group M (P < 0.001 and P < 0.001, respectively). In addition, a significant difference in ET_SEVO_ was found between Group MP3 and Group MP6 (P = 0.003). The HR, SAP, DAP, and MAP values during maintenance anesthesia in the three groups are shown in Table 1. There were no significant differences among the groups. The MAP values were maintained over 70 mmHg throughout the maintenance period in all groups. However, the mean dobutamine infusion rates required to maintain the MAP over 70 mmHg in Group MP6 (0.15 ± 0.06 μg/kg/min) and Group MP3 (0.20 ± 0.10 μg/kg/min) were significantly lower than those required in Group M (0.37 ± 0.18 μg/kg/min) (P < 0.001 and P = 0.007, respectively). The mean values of PaCO_2_ were 49 ± 2 mmHg in Group M, 52 ± 4 mmHg in Group MP3, and 50 ± 5 mmHg in Group MP6. There were no significant differences among the groups. The mean values of PaO_2_ were 494 ± 56 mmHg in Group M, 433 ± 97 mmHg in Group MP3, and 488 ± 66 mmHg in Group MP6; there were no significant differences among groups.

**Table 1:** Cardiovascular parameters during maintenance of anesthesia

Variable	Group	Time after connection to breathing circuit (min)				
		0	15	30	45	60
HR (beats/min)	M	–	26 ± 2	25 ± 4	26 ± 2	27 ± 1
	MP3	–	28 ± 3	29 ± 2	29 ± 2	30 ± 2
	MP6	–	28 ± 4	29 ± 4	29 ± 4	29 ± 3
SAP (mmHg)	M	–	102 ± 11	95 ± 10	104 ± 10	108 ± 6
	MP3	–	104 ± 8	103 ± 9	105 ± 8	108 ± 6
	MP6	–	111 ± 15	106 ± 10	109 ± 8	113 ± 8
DAP (mmHg)	M	–	65 ± 10	55 ± 12	60 ± 6	64 ± 5
	MP3	–	63 ± 5	62 ± 4	63 ± 5	68 ± 4
	MP6	–	67 ± 12	68 ± 7	71 ± 6	74 ± 7
MAP (mmHg)	M	–	78 ± 10	71 ± 9	75 ± 5	77 ± 3
	MP3	–	78 ± 5	77 ± 3	78 ± 4	81 ± 6
	MP6	–	82 ± 13	82 ± 8	85 ± 6	87 ± 7

Data are presented as mean ± SD

Horses were maintained with sevoflurane and CRI of either medetomidine (3.0 μg/kg/h) (Group M; n = 10); or medetomidine (3.0 μg/kg/h) and propofol (3.0 mg/kg/h) (Group MP3; n = 10); or medetomidine (3.0 μg/kg/h) and propofol (6.0 mg/kg/h) (Group MP6; n = 10)

None of the data across rows differed significantly from each other

*HR* heart rate, *SAP* systolic arterial blood pressure, *DAP* diastolic arterial blood pressure, *MAP* mean arterial blood pressure

The plasma propofol concentrations after induction, during maintenance, and after standing in Group MP3 and Group MP6 are shown in Table 2. The plasma propofol concentration during maintenance was calculated to be 1.1 ± 0.2 to 1.2 ± 0.2 μg/mL in Group MP3 and 2.4 ± 0.4 to 3.0 ± 0.4 μg/mL in Group MP6. Thus, the values in Group MP3 were stable and did not change significantly throughout maintenance. On the other hand, those in Group MP6 were significantly greater after 45 min of maintenance than at 15 min (P < 0.01). Moreover, the propofol concentration at each time point during maintenance was significantly greater in Group MP6 than in Group MP3. The plasma propofol concentrations after standing in Group MP3 and Group MP6 (0.4 ± 0.3 μg/mL, 0.7 ± 0.4 μg/mL, respectively) were significantly lower than those during maintenance (P < 0.01 and P < 0.01, respectively).

**Table 2:** Plasma propofol concentrations after induction of anesthesia, during sevoflurane maintenance, and after standing

Group	After induction (µg/mL)	Time after connection to breathing circuit (min)			After standing (µg/mL)
		15 (µg/mL)	30 (µg/mL)	45 (µg/mL)	
MP3	0.7 ± 0.2^A^	1.1 ± 0.2^B^	1.2 ± 0.2^B^	1.2 ± 0.2^B^	0.4 ± 0.3^C^
MP6	0.6 ± 0.3^A^	2.4 ± 0.4^B^*	2.7 ± 0.3^BC^*	3.0 ± 0.4^C^*	0.7 ± 0.4^AD^

Data are presented as mean ± SD

Horses were maintained with sevoflurane and CRI of either medetomidine (3.0 μg/kg/h) and propofol (3.0 mg/kg/h) (Group MP3; n = 10) or medetomidine (3.0 μg/kg/h) and propofol (6.0 mg/kg/h) (Group MP6; n = 10)

Data with the same superscript letters within the same row do not differ significantly from each other

* Significant difference from Group MP3 (P < 0.05)

**Fig. 2:**
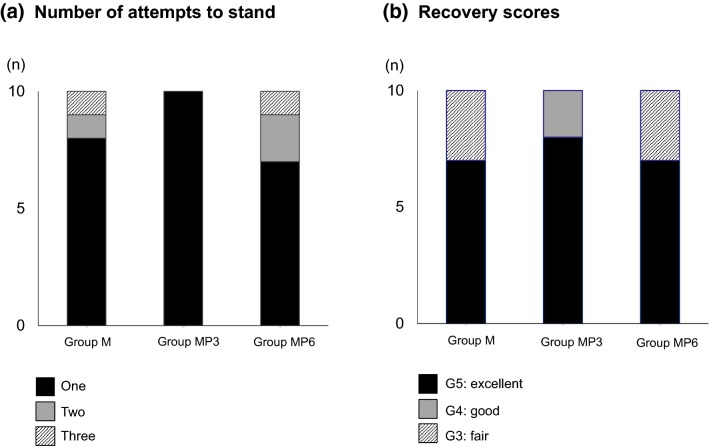
Number of attempts to stand (**a**) and recovery scores (**b**). Horses were maintained with sevoflurane and CRI of either medetomidine (3.0 μg/kg/h) (Group M; n = 10); or medetomidine (3.0 μg/kg/h) and propofol (3.0 mg/kg/h) (Group MP3; n = 10); or medetomidine (3.0 μg/kg/h) and propofol (6.0 mg/kg/h) (Group MP6; n = 10)

The number of attempts to stand and the recovery scores are shown in Fig. 2. There were no significant differences among groups; notably, all horses in Group MP3 required only one attempt to stand, and the quality of their recovery was excellent. The mean recovery times at appearance of spontaneous respiration, extubation, first movement, sternal recumbency, first attempt to stand, and standing in the three groups are shown in Table 3. There were no significant differences among the groups.

**Table 3:** Times from end of anesthesia to recovery events

Group	Recovery event					
	Appearance of spontaneous respiration (min)	Extubation (min)	First movement (min)	Sternal recumbency (min)	First attempt to stand (min)	Standing (min)
M	8 ± 6	14 ± 8	32 ± 7	36 ± 6	40 ± 6	40 ± 6
MP3	10 ± 5	17 ± 6	36 ± 11	39 ± 12	41 ± 11	41 ± 11
MP6	12 ± 5	17 ± 5	36 ± 8	42 ± 10	46 ± 14	47 ± 14

Data are presented as mean ± SD

Horses were maintained with sevoflurane and CRI of either medetomidine (3.0 μg/kg/h) (Group M; n = 10); or medetomidine (3.0 μg/kg/h) and propofol (3.0 mg/kg/h) (Group MP3; n = 10); or medetomidine (3.0 μg/kg/h) and propofol (6.0 mg/kg/h) (Group MP6; n = 10)

None of the data across rows differed significantly from each other

## Discussion

We demonstrated that co-administration of medetomidine–propofol CRI during maintenance significantly reduced the sevoflurane requirement for surgical anesthesia in Thoroughbred racehorses, and that the sevoflurane requirement was significantly lower with the higher propofol infusion rate than with the lower one. Propofol, like inhalation anesthetics, directly depresses dorsal horn neuronal responses to noxious mechanical stimulation [20]. The potent anesthetic effect of propofol likely compensates for the reduction of the sevoflurane concentration. Propofol administration at a dose similar to the one we used (3.0 mg/kg/h) decreases the desflurane MAC by 28% in ponies [21] and the isoflurane end-tidal concentration by 20% in horses [22]. Villalba et al. [18] achieved a 65% reduction in the MAC of isoflurane with the administration of propofol (3.0 mg/kg/h) and medetomidine (1.25 μg/kg/h) CRI. In goats, propofol reduces isoflurane requirements by 16% at a dose of 3.0 mg/kg/h and by 34% at 6.0 mg/kg/h [23]. As in the previous study [18] we were unable to determine the individual effects of sevoflurane, propofol, and medetomidine; however, the different pharmacological interactions of the different combinations of drugs used may have caused the differences in requirements for inhalation anesthetic.

We found here that the mean plasma propofol concentrations were 1.1–1.2 μg/mL with a loading dose of 1.0 mg/kg followed by a CRI of 3.0 mg/kg/h, and 2.4–3.0 μg/mL with the same loading dose followed by a CRI of 6.0 mg/kg/h. We considered that these plasma concentrations caused significant reductions in the sevoflurane requirement. However, with CRI at 6.0 mg/kg/h the plasma propofol concentration increased significantly during the maintenance. Therefore, because accumulation of propofol or its active metabolites may occur during long-term anesthesia, we recommend propofol CRI at 3.0 mg/kg/h rather than 6.0 mg/kg/h in clinical cases.

The pharmacokinetics of CRI of propofol in horses have already been studied [24]; however, the plasma propofol concentrations required to provide anesthetic effects during sevoflurane anesthesia in horses had not, to our knowledge, been determined before our study. In goats, propofol CRI at 3.0 mg/kg/h reduces the isoflurane MAC by 16% and propofol CRI at 6.0 mg/kg/h reduces the isoflurane MAC by 34% [23]. The corresponding reported plasma concentrations of propofol were 1.2–1.8 μg/mL and 2.3–3.0 μg/mL, respectively [23], quantitatively similar to the plasma concentrations we obtained. Plasma propofol concentrations have also been reported to be 2.0–4.7 μg/mL at doses of 12 mg/kg/h in sheep [25]; 2.3–6.5 μg/mL at 9–12 mg/kg/h in ponies [14]; and 3.8–5.8 μg/mL at 24 mg/kg/h in dogs [26]. The doses of propofol used in these previous studies differ slightly from those that we used here; therefore, the reason for the difference in plasma concentrations between our results and those of these studies cannot be determined from this study and requires further investigation. Differences in propofol metabolism among species or differences in the pharmacological interactions of the different combinations of drugs used may have caused the differences in propofol plasma concentrations.

Propofol decreases arterial blood pressure in a dose-dependent manner through a direct vasodilator effect [27, 28]. However, this effect may be reduced when propofol is combined with medetomidine, most likely because the vasoconstriction caused by medetomidine counteracts the propofol-induced vasodilatation [29, 30]. Some authors have suggested that TIVA with medetomidine–propofol is associated with adequate maintenance of cardiovascular function and a reduction in the dobutamine requirement in horses [31–33]. On the other hand, the combination of medetomidine and propofol CRI in a previous study resulted in an increase in MAP [18]. Inhalational anesthetics are potent dose-dependent depressors of cardiovascular function [34, 35]; therefore, the improved arterial blood pressure and lower dobutamine requirements observed when the medetomidine–propofol CRI was employed in this study may have been a consequence of the reduction in sevoflurane requirements. Nevertheless, we found no significant differences in the dobutamine requirements between horses given CRI of medetomidine (3.0 μg/kg/h) and propofol (3.0 mg/kg/h) and those given medetomidine (3.0 μg/kg/h) and propofol (6.0 mg/kg/h). Thus, small to moderate amounts of positively inotropic agents were needed during co-administration of medetomidine and propofol because the level of improvement of cardiovascular depression was moderate during maintenance. Additionally, use of the medetomidine–propofol CRI anesthetic protocol seemed to have a plateauing effect on dobutamine requirements, but further studies are needed with different doses of propofol to demonstrate this more clearly.

Good quality recovery from anesthesia is important in horses, as in other species, and it depends on a number of factors, including, but not limited to, the sedative and anesthetic drugs used, the duration of anesthesia, the degree of postoperative pain, the horse’s temperament, and any limitations to standing caused by surgery or anesthesia-induced myopathy or neuropathy [36, 37]. Both propofol and medetomidine have been associated with a good quality of recovery. In horses, propofol produces high-quality anesthetic recoveries that are smooth and calm in the attempt to stand [38, 39]. Alpha-2 adrenoceptor agonists administered at the end of anesthesia improve the quality of recovery in horses [40] and, when compared with lidocaine, medetomidine is considered to provide better recoveries following sevoflurane anesthesia [11]. We found here that propofol administered at a loading dose of 1.0 mg/kg followed by CRI at 3.0 mg/kg/h gave the best recovery quality. We were unable to find any previous studies in the available literature on the relationship between propofol plasma concentration and time to recovery from inhalation anesthesia. Boscan et al. noted that plasma propofol concentration appeared to be correlated with recovery quality and that better recoveries were associated with lower propofol plasma concentrations [24]. When our horses stood, their mean plasma propofol concentrations were approximately 0.4 μg/mL for horses given CRI of medetomidine (3.0 μg/kg/h) and propofol (3.0 mg/kg/h) and 0.7 μg/mL for those given medetomidine (3.0 μg/kg/h) and propofol (6.0 mg/kg/h). There were no significant differences in the number of attempts to stand or in recovery scores between the two infusion rates; however, the differences in plasma propofol concentrations after standing might have had a non-significant effect on recovery quality.

## Conclusions

Compared with a CRI of medetomidine alone, CRIs of medetomidine and propofol reduced the sevoflurane requirement for surgical anesthesia as the propofol dose increased; additionally, it provided good maintenance of cardiovascular function. Specifically, CRI of medetomidine (3.0 μg/kg/h) and propofol (3.0 mg/kg/h) produced excellent recovery quality and could be an especially useful addition to sevoflurane anesthesia in Thoroughbred racehorses undergoing arthroscopic surgery.

## Abbreviations

ANOVA: analysis of variance
CRI: constant rate infusion
DAP: diastolic arterial blood pressure
ET_SEVO_: end-tidal sevoflurane concentrations
HR: heart rate
IV: intravenous
MAP: mean arterial blood pressure
P: probability
PaCO_2_: arterial carbon dioxide tension
PaO_2_: arterial oxygen partial tension
SAP: systolic arterial blood pressure
SD: standard deviation
